# Minority stress theory applied to conception, pregnancy, and pregnancy loss: A qualitative study examining LGBTQ+ people’s experiences

**DOI:** 10.1371/journal.pone.0271945

**Published:** 2022-07-26

**Authors:** Ashley Lacombe-Duncan, Nazanin Andalibi, Lee Roosevelt, Emma Weinstein-Levey

**Affiliations:** 1 University of Michigan School of Social Work, Ann Arbor, Michigan, United States of America; 2 University of Michigan School of Information, Ann Arbor, Michigan, United States of America; 3 Department of Health Behavior and Biological Sciences, University of Michigan School of Nursing, Ann Arbor, Michigan, United States of America; Virginia Commonwealth University, UNITED STATES

## Abstract

Many lesbian, gay, bisexual, transgender (trans), queer, and other sexual and gender minority (LGBTQ+) people desire to conceive children. Yet, LGBTQ+ peoples’ experiences are scant in reproductive health literature, particularly around pregnancy loss—a stigmatized and distressing pregnancy outcome. Informed by minority stress theory, this qualitative study aimed to explore the experiences of multi-level stigma and resilience among LGBTQ+ people in the context of conception, pregnancy, and loss. Seventeen semi-structured individual interviews (25–70 minutes) were conducted (2019) with a purposive sample of LGBTQ+ people in the United States (U.S.) who had experienced pregnancy loss (n = 14) or in an intimate partnership in which a pregnancy was lost (n = 3) in the last two years. Transcribed interviews were analyzed thematically. Participants described the profound sadness of pregnancy loss due to unique challenges of LGBTQ+ conception. Multiple types of stigma manifested at intrapersonal (e.g., anticipated sexual stigma upon disclosure), interpersonal (e.g., unsolicited advice about conception decisions), and structural levels (e.g., differential requirements to access conception compared to heterosexual/cisgender couples). Resilience was also seen individually (e.g., purposeful disclosure of conception, pregnancy, and loss), relationally (e.g., connecting with other LGBTQ+ community members), and collectively (e.g., creating/engaging in LGBTQ+-specific conception, pregnancy, and loss online spaces). LGBTQ+ people experience minority stressors of multi-level stigmatization throughout the pregnancy process, which limits their access to social support after experiencing pregnancy loss. However, individual, relational, and collective resilience strategies abound in response. Thus, minority stress theory can also be applied to recognize strengths-based and affirming approaches to reproductive healthcare for LGBTQ+ people.

## Introduction

Many lesbian, gay, bisexual, transgender (trans), queer, and other sexual and gender minority (LGBTQ+) people desire to conceive children [1, 2]. A devastating outcome of some pregnancies is pregnancy loss. While there is no population-level epidemiological research documenting the prevalence of pregnancy loss among LGBTQ+ people broadly, one study utilizing data from the United States (U.S.) 2006–2015 National Survey of Family Growth identified pregnancy loss prevalence rates of 32.7% and 34.2% among bisexual and lesbian women, respectively [3]. These rates appear much higher than the Centers for Disease Control and Prevention (CDC) estimate of approximately 10–20% of recognized pregnancies ending in pregnancy loss [4]. LGBTQ+ people’s experiences have been understudied in the context of conception, pregnancy, and, most critically, pregnancy loss, despite documented significant sexuality and gender inequities in pregnancy and birth outcomes [3, 5, 6]. These differences are hypothesized to be due to lower access to health care and additional stress related to sexual and anti-trans stigma from providers and institutions, as well as resultant negative chronic physical health and mental health outcomes, such as depression [3, 5, 6].

Irrespective of gender or sexual orientation, the psychological impacts of pregnancy loss are deep and myriad [7], including profound sadness and depression [6, 8], stress and anxiety [9], and post-traumatic stress disorder [9, 10]. Those who physically experience the loss may also experience guilt and self-blame [8, 11–13]. The unique stressors surrounding the assisted reproductive technology (ART) process [14, 15], which is already associated with depression and anxiety among sexual minority women [16], may exacerbate mental health struggles in the context of loss [17]. Pregnancy loss may be viewed as an acute stressor, while pregnancy loss stigma is ongoing and perpetuated within the broader system of patriarchy which bases a cisgender woman’s worth in her capacity to have biological children [18, 19].

The pervasiveness and insidiousness of pregnancy loss stigma has been well-documented [12, 13, 20]. For example, results of a cross-sectional survey showed that almost half of participants (49%) experienced stigma at the time of loss and over one-quarter (27%) during follow-up care, while almost one-quarter (23%) noted that pregnancy loss stigma was a barrier to support-seeking [20]. Most prevalently discussed was interpersonal stigma by healthcare providers (e.g., insensitive language). However, lack of recognition of the experience of pregnancy loss in the workplace—a manifestation of structural stigma—also had significant consequences for participants [20]. Through review of 23 articles, Pollock and colleagues [12] further conceptualized both public (interpersonal) and self- (internalized) stillbirth stigmas. Interpersonal stigma was characterized by a minimization of pregnancy loss by others, loss of friends or family when discussing stillbirth, and silencing when discussing their stillborn baby. Similar to the study by Watson et al. [20] Pollock et al. [12] also identified stigmatizing healthcare providers, with severe manifestations present during stillbirth including being left to labor alone and having no access to pain medication. Self-blame, shame, and guilt were common. This internalized stigma was also evidenced in Murphy’s qualitative study documenting mothers’ attempts to distance themselves from stigmatized health behaviors, such as smoking [13]. While some literature has documented experiences of pregnancy loss among LGBTQ+ people [21–23], the intersections of pregnancy loss stigma and sexual or anti-trans stigma have been under explored, particularly through the lens of minority stress theory.

Indeed, minority stress theory has been posited to explain how people with marginalized identities experience chronic and cumulative stress. More specifically, minority stress theory explains how sexual and anti-trans stigma related to sexual and gender minority status, respectively, contribute to psychological distress [24–27] and negative physical health outcomes [28] among LGBTQ+ people. Sexual and anti-trans stigma disrupt opportunities for health-promoting behaviors, limit access to social support and community ties, and, ultimately, cause biological wear and tear on the body by increasing cortisol production and allostatic load [29, 30]. Marginalization and stigma are related concepts. Stigma refers to social and institutional processes and structures that devalue marginalized individuals and communities, such as LGBTQ+ people, and limit their access to power and opportunities [25, 31]. Stigma is further conceptualized as operating at the level of the individual, or proximally, as well as at interpersonal and structural levels more distally [24, 32].

Research published to-date has focused on the experiences of lesbian and bisexual cisgender women have identified experiences of heteronormative reproductive healthcare, including structural stigma, such as a lack of inclusive forms [21, 23, 33–35]. Heteronormativity “assumes that heterosexuality is the indisputable and unquestionable bedrock of society” and reduces all other forms of sexual expression to “pathological, deviant, invisible, unintelligible, or written out of existence” (p.167) [36]. Another manifestation of structural stigma is lack of medical insurance or coverage of ART, which contributes to stress among LGBTQ+ people seeking parenthood [23, 35, 37]. Cisnormativity, defined as the “sociocultural assumptions and expectations that all people are cissexual and/or have a cisgender body” (p. 356) [38], is also evident in the critical research and practice gaps seen in reproductive healthcare for trans and gender diverse people [39]. Interpersonal stigma is also pervasive among healthcare providers, such as discriminatory comments and cisnormative and heteronormative language (e.g., utilizing gendered and/or misgendering language when referring to parents) [21, 23, 40]. There are many negative consequences of such interpersonal stigma from healthcare providers, such as avoidance of care [41] and concealment of one’s identities [14], further exacerbating negative impacts of minority stress. Finally, stigmatizing experiences have been found to influence resultant psychological distress during pregnancy among trans men [42] and pregnancy loss among sexual minority women[14] and trans men and nonbinary people [6].

Resilience has been identified as an essential component of minority stress theory [43–45], as it plays a role in determining how LGBTQ+ people are impacted by stigmatizing experiences. Resilience has been defined as “both the capacity of individuals to navigate their way to the psychological, social, cultural, and physical resources that sustain their wellbeing, and their capacity individually and collectively to negotiate for these resources to be provided and experienced in culturally meaningful ways” [46] (p. 225). In this way, similarly to stigma, resilience is conceptualized as a multi-dimensional construct involving individual, relational, and collective elements [46–49], combatting an individualistic, personal responsibility, view [45].

Relational and collective resilience may be particularly important for fostering health among LGBTQ+ people. For example, in one study [50], seeking social support—a form of relational resilience—was negatively associated with perceived stress and allostatic load among 46 cisgender LGB people [50]. Prior work has shown that LGBTQ+ communities foster relational and collective resilience in both in-person and online through, for example, social media [51, 52]. Studies conducted with cisgender heterosexual women have also documented the utility of online spaces to build relational resilience among those who have experienced pregnancy loss [53]. Moreover, Craven’s [23] recently published book entitled Reproductive Losses has one chapter dedicated to queer resiliency, in which she notes the evidence of resilience in response to loss through individual coping strategies, community support, and commemoration. Yet, scant literature has applied the multi-dimensional construct of resilience to understand how LGTBQ+ people navigate stigma during conception and pregnancy, and particularly to how LGTBQ+ people respond to pregnancy loss in offline nor online spaces. A multi-level resilience lens that takes into consideration multiple contexts of expression has the potential to shift to a strength-based and empowering discussion of reproductive health for LGBTQ+ people.

Thus, informed by minority stress theory, the objective of this qualitative study was to explore the experiences of stigma and resilience among LGBTQ+ people in the context of conception, pregnancy and pregnancy loss across online and offline spaces.

## Materials and methods

This exploratory qualitative study guided by principles of thematic analysis aimed to examine LGBTQ+ people’s disclosure and social support needs, challenges, and social technology (e.g., social media) use/non-use in relation to pregnancy loss, with a specific emphasis on understanding how multiple intersecting stigmatized identities/experiences inform disclosure and support seeking decisions and outcomes.

### Recruitment and sampling

In April 2019 we conducted semi-structured individual interviews with participants who: a) self-identified as LGBTQ+; b) had experienced pregnancy loss or been in an intimate partnership in which a pregnancy was lost in the last two years; c) had used any type of social media; d) lived in the U.S.; and e) were over the age of 18. The decision to include non-gestational partners of those who had experienced pregnancy loss was made to recognize the secondary or vicarious stigma non-gestational parents may experience [54, 55] and to honor the complex and nuanced decision-making process LGBTQ+ couples undergo to decide who will carry a pregnancy and thereby the differential impacts of loss on non-gestational LGBTQ+ parents as compared to non-gestational cisgender and heterosexual partners.

Participants were recruited via individual social media networks of the principal investigators (ALD, NA, LR) (e.g., Facebook, Twitter) as well as through relevant Facebook groups the investigators were a part of and through word-of-mouth. These relevant Facebook groups included the largest LGBTQ+ parenting group on Facebook, ‘LGBTQ+ Pregnancy to Parenting’ (approximately 6000 members) and an offshoot of that group called ‘LGBT+ Trying to Conceive & Parents Group’ (approximately 4500 members), among others. Interested participants completed a brief (5-minute) screening and sociodemographic survey. A total of 44 complete responses to the screening survey were collected, 6 of which did not meet study criteria. Thirty-five of the remaining 38 participants were contacted for interview, 17 of whom responded, completed the online consent form, scheduled an interview, and completed an interview. Attrition of the remaining 18 potential participants occurred at various points (non-response to initial invitation, non-completion of consent/schedule of interview, no show to interview).

Of the 17 participants, 14 (82.4%) identified as having physically lost a pregnancy and 3 (17.6%) as having been in an intimate partnership in which a pregnancy was lost (Table 1). Almost one-quarter of participants (n = 4; 23.5%) reported the pregnancy loss occurred within the three months preceding the interview. Participants were a mean age of 34.4 (standard deviation [SD]: 3.3). The majority identified as cisgender women (n = 15; 88.2%), with one participant identifying as transmasculine and one participant identifying as non-binary. Participants identified with a wide variety of sexual orientations. Most were white (n = 13; 76.4%), married (n = 16; 94.1%), with a graduate degree (n = 13; 76.4%), income $50 000 or above (n = 14; 82.4%), and living in urban settings (n = 14; 82.4%).

**Table 1:** Participant characteristics.

Factors	Mean (SD) or N (Proportion)^a^
**Pregnancy loss experiences**	
Pregnancy loss experience	
Physically experienced pregnancy loss	14 (82.4)
In an intimate partnership in which	3 (17.6)
pregnancy loss occurred	
Year in which pregnancy loss occurred	
2019	4 (23.5)
2018	8 (47.1)
2017	5 (29.4)
**Sociodemographic characteristics**	
Age	34.4 (3.3), Range: 29 to 40
Gender identity	
Cisgender woman	15 (88.2)
Transmasculine person	1 (5.9)
Non-binary person	1 (5.9)
Sexual orientation	
Lesbian	3 (17.6)
Bisexual	1 (5.9)
Queer	5 (29.4)
Asexual (biromantic, demiromantic)	2 (11.8)
More than 1 sexual orientation (e.g.,	6 (35.3)
Lesbian/Queer, Bisexual/Queer)	
Race/ethnicity (n = 16)	
White	13 (76.4)
Black/African American	1 (5.9)
Latinx	1 (5.9)
Multiple races/ethnicities	1 (5.9)
Relationship	
Married	16 (94.1)
Single	1 (5.9)
Education	
College	4 (23.5)
Graduate Degree	13 (76.4)
Income	
$1,000-$29,999	2 (11.8)
$30,000-$49,999	1 (5.9)
$50,000-$74,999	4 (23.5)
$75,000+	10 (58.8)
Geography	
Urban	14 (82.4)
Rural	3 (17.6)

^a^ Sample size n = 17 unless otherwise noted.

### Procedures

Participants were interviewed virtually over a communication tool of their choice (e.g., Skype). Virtual interviewing allowed for inclusion participants from across widely dispersed geographic locales as well as greater control of participant’s over their anonymity and ability to complete the interview in a space most comfortable to them [53].

Interviews lasted between 25 and 97 minutes (average 67 minutes, standard deviation 21 minutes) and were facilitated by the use of an interview guide (one for those who had physically experienced pregnancy loss and one for those who had been in an intimate partnership in which a pregnancy was lost). The interview guides explored participant narratives (e.g., “What was your life like when you wanted to become pregnant/become a parent with your partner, and when you found out you were pregnant/expecting?”), online and offline disclosure of pregnancy loss (e.g., “Did you tell anyone about your experiences using any type of social media?”), gaps/needs after experiencing pregnancy loss (e.g., “What were/are 2–5 things that you most needed/need afterwards that would have/would help you process your experience?) and social support desires (e.g., “What did/does/would an ideal support network for you in relation to pregnancy loss look like?”).

Interviews were audio-recorded and transcribed verbatim. Participants completed an online informed consent form. Those who completed the interview received a $25 honorarium. All study procedures were approved by the University of Michigan Institutional Review Board (HUM00159413). The study was considered exempt and not regulated. Participants completed an informed consent process whereby they reviewed an electronic consent form and indicated their willingness to participate by checking a box.

### Data management and analysis

We applied a thematic approach to data analysis to explore inductively-generated themes utilizing an iterative and reflexive process [56–58]. We followed Braun & Clarke’s [58] six steps of thematic analysis, drawing on Attride-Stirling’s [56] in-depth description of extracting and organizing themes and Fereday & Muir-Cochrane’s [57] example of rigor in thematic analysis. First, two team members (ALD and EWL) read and re-read the transcripts to familiarize themselves with the data. Then, ALD and EWL independently open-coded six transcripts, meeting together three times to generate an overarching initial code list. Next, codes were categorized under overarching themes: Context, and then consistent with multi-level stigma and resilience theories [24, 32, 46–49], Intrapersonal Stigma, Interpersonal Stigma, Structural Stigma, Individual Resilience, Relational Resilience, and Collective Resilience. Then, EWL continued coding the remainder of the transcripts, meeting three additional times with ALD to discuss the coding process and any codes that did not fit within the preliminary overarching themes. New codes were generated until data saturation was reached, whereby a transcript generated no new codes. Codes and illustrative quotes were combined by EWL into seven documents with each several sub-themes, along with a brief interpretation. Consistent with the fourth step from Braun & Clarke [58], ALD then reviewed all seven documents, further synthesizing a conceptual framework of multi-level stigma and resilience in the context of conception, pregnancy, and loss. Finally, NA and LR reviewed the analysis, providing additional interpretation from their experience of conducting the interviews, pregnancy loss-related scholarship and diverse lived experiences in relation to the topic, exemplifying personal reflexivity [59], before a final write-up was produced. Both independently and through multiple conversations throughout the research process—from study conceptualization to manuscript generation—we reflected on our own experiences as members of the LGBTQ+ community including those who have experienced pregnancy loss. We also reflected on our professional roles (nurse midwife clinician researcher, social work clinician researcher, human computer interaction researcher) and shared passion of promoting access to intersectionally-affirming care for LGBTQ+ people in both online and offline spaces. We also engaged in a processes of critical reflexivity, which necessitated integrating content on the current socio-political climate affecting LGBTQ+ people in the U.S. and contextualizing our findings through this lens [59]. Other commonly deployed strategies for enhancing rigor of the analysis were utilized, including peer debriefing between members of the research team and with audience members during conference presentations, and negative case analysis whereby discrepant findings were sought through the analysis and highlighted in the results [60–63].

## Results

Findings showed that LGBTQ+ people experience minority stressors of multi-level stigmas at intrapersonal, interpersonal, and structural levels during pregnancy and pregnancy loss, including sexual stigma, anti-trans stigma, and pregnancy loss stigma. However, even in the face of such stigmas and the profound devastation of pregnancy loss, participants expressed individual, relational, and collective resilience, which helped to reduce the negative impact both of stigma and pregnancy loss. Results first address the broad context of LGBTQ+ conception, pregnancy, and loss, followed by multi-level stigma (intrapersonal, interpersonal, and structural) and multi-level resilience (individual, relational, and collective). Exemplary quotes are embedded throughout, with participant characteristics noted at first quotation. Additional quotes are presented in Tables 2–4.

**Table 2:** Broad context of LGBTQ+ conception, pregnancy, and loss sub-themes, definitions, and quotes.

Theme	Example Quotes
Fear of pregnancy loss (n = 4)	“I was really on edge when I found out I was pregnant again, but I was trying to be present with that I was pregnant and trying to be positive…. I’m in a place where I’m again, still on edge about the possibility of having a miscarriage but then I’m also on edge about just not having it take at all.” (P14, 30s, Latinx, Queer, Cisgender)
	“The hardest part was, there is, between things happening, there’s just a lot of uncertainty.” (P11, 30s, White, Lesbian/Queer, Cisgender)
Profound sadness (n = 12)	“I was just feeling bewildered and lost…. I was in a place where it was just like brushing my hair was really difficult.” (P4, 30s, White, Asexual/Biromantic, Cisgender)
	“I still feel sad about it. I still feel like I’m hyper aware of how things would be different in our lives if that pregnancy had continued…. It was a really long, drawn out, terrible process.” (P12, 30s, White, Queer, Cisgender)
Challenges of conceiving while LGBTQ+ (n = 11)	“When you’re a queer person doing this, you have to talk to someone about it, whether that’s because you have to ask your friend for their sperm or it’s because you have to go to a doctor and say, ‘Can you do this procedure for me?’…. The intentionality and the effort that goes into the process of getting pregnant is different…. It just sort of feels like we’ve had to really fight for this.” (P7, 30s, White, Queer/Lesbian/Gay, Cisgender)
	“Nine times out of 10, especially if the queer couple is making children together, nine times out of 10, it wasn’t just a, ‘Here’s your boyfriend, here’s your girlfriend, now you’ve made a baby, and you didn’t have to plan it out, and you didn’t have to make this very specific decision.” (P17, 30s, Partner, Unidentified race, Queer, Cisgender)

**Table 3:** Multi-level stigma in LGBTQ+ people’s experiences of conception, pregnancy, and loss: Sub-themes, definitions, and quotes.

Intrapersonal Stigma (n = 11)	
Anticipated and internalized sexual stigma (n = 3)	“I don’t want to give people anymore ammunition that they already have…. Or even just feelings towards LGBTQ couples in general. If we’re the only ones that they know, and we’re having trouble having kids then they could generalize that to, ‘Well, that’s because LGBTQ shouldn’t have kids.’” (P8, 30s, Asian and White, Asexual and Demiromantic, Cisgender)
	“Obviously all of the ‘They’re going to make their kids gay.’ You hear those kinds of stories and things that are out there. Again, luckily, I didn’t experience any of that myself. But it certainly is in my mind, knowing that that is part of people’s experience.” (P7, 30s, White, Queer/Lesbian/Gay, Cisgender)
Internalized infertility and/or pregnancy loss stigma (n = 8)	“I did it to myself, too, of like, ‘well what could I have done differently’ or ‘what did I do wrong,’ or whatever.’” (P16, 40s, White, Queer, Cisgender)
	“…We were really excited, a little uncautious. We told a lot of people. We were just so over the moon at the idea….” (P6, 30s, Partner, White, Hispanic, Lesbian, Transmasculine)
Interpersonal Stigma (n = 14)	
Being asked inappropriate/invasive questions (n = 5)	“And when we told everyone we were pregnant the first time around, she [a family member] was literally just like, baffled. She was like, how did this happen? I mean she asked us if it was an accident, and my wife was like, like what? I don’t even know how to answer the question.” (P12, 30s, White, Queer, Cisgender)
	“He’s [retired medical provider church member] the one asking the questions. I think the last time he asked at least… my takeaway from the way he asked the question was he was trying to communicate to us that IVF is still an option…. So maybe he’s supportive, I don’t know.” (P8, 30s, Asian and White, Asexual and Demiromantic, Cisgender)
Unsolicited advice/judgment about conception and pregnancy process (n = 5)	“… She [reproductive endocrinologist] was grilling us on all this stuff about our donor and very flippantly was like, ‘Okay, well, would you use a different donor?’ I was like, “No.” Essentially asking me would I choose a different spouse. This is a really intentional choice. We had really thought about the way we wanted to make our family. To have someone suggest that it would be okay to us to just swap that out for someone else meant to me that they didn’t understand the emotional component of how we had decided to go about this.” (P7, 30s, White, Queer/Lesbian/Gay, Cisgender)
	“My sister did say something like, ‘Well, you know, why are you guys even trying to do biological kids? Why not just adopt?’ And I was pretty offended. I was like, ‘Why didn’t you?’ People can choose to have biological children regardless of their sexual orientation…. So, I felt like there was this different standard for me, and that I shouldn’t even want this….” (P15, 30, Partner, White, Lesbian, Cisgender)
Cisnormative and/or heteronormative assumptions (n = 8)	“I’ve heard a lot of people’s experiences in the queer parent’s group on Facebook, kind of around that. When they go to parental classes and birthing classes and stuff like that…they use incredibly gendered, heteronormative language like, mother and a father.” (P15, 30, Partner, White, Lesbian, Cisgender)
	“…. I’m non binary trans, and find gendered language talking about pregnancy pretty upsetting and any group that isn’t specifically and explicitly LGBTQ and welcoming is going to use language of women and mothers, even if they say oh yeah, we’re fine with queer people, but they’re still gonna talk about women and mother constantly.” (P3, 30s, White, Queer/Bi/Pan, Nonbinary)
Intersecting interpersonal stigmas (n = 4)	[intersecting sexual and relationship status stigma] “The thing that’s missing in those majority straight groups is that there is this presumption, and this happens in the queer trying to conceive group too to some extent, but there’s this presumption there’s a partner….” (P13, 40s, White, Bisexual/Queer, Cisgender)
	[intersecting sexual stigma and racism] “So I had noticed about a week before that I was having some leaking and I called the nurse about it…. And I got kind of dismissed, like, ‘Oh you’re probably just peeing.’ And I was like, ‘I don’t think that it’s urine but it’s possible, I don’t know….” (P1, Black, Bisexual, Cisgender)
Structural Stigma (n = 11)	
Cisnormativity and heteronormativity embedded in systems (e.g., pregnancy apps, healthcare system) (n = 4)	“I definitely didn’t use any social networking applications on [Ovia] or any other pregnancy sites because I found those to be really, really heteronormative. I don’t know, I just didn’t feel like I belonged there.” (P5, 20s, White, Lesbian, Cisgender)
Lack of LGBTQ+-specific services and resources, particularly intersectionally-affirming (n = 4)	“I would have really liked to have had a book, or like something to read about how queer people had overcome their experiences of miscarriage. That would have been helpful to me because I did have some normalizing things for heterosexual people, and they were helpful to a degree. But it would have just been more helpful to have people who felt like me.” (P5, 20s, White, Lesbian, Cisgender)
	“Making sure that you’re including people of all different abilities, all different ethnicities, all different sexual orientations, all different gender identities. Have families who look different because families look different.” (P1, Black, Bisexual, Cisgender)
Cost/lack of coverage (n = 6)	“… But every time we’re like, I don’t wanna spend another 500 dollars…You know. So we were really hoping that this pregnancy would, and then, because we’re, hopefully after this one we’re done.” (P17, 30s, Partner, Unidentified race, Queer, Cisgender)
	“So just thinking about like if we depleted all of that money again and then didn’t have any it was like, “What do we do?”” (P1, Black, Bisexual, Cisgender)

Any type of stigma endorsed by n = 17; All types of stigma endorsed by n = 6.

**Table 4:** Multi-level resilience in LGBTQ+ people’s experiences of conception, pregnancy, and loss: Sub-themes, definitions, and example quotes.

Individual Resilience (n = 17)	
Attending to one’s emotions in response to pregnancy loss (n = 12)	“I was actually out of town at work training when my period started and I was super disappointed and went and bought myself some greasy food and a pack of cigarettes and had a nice cry that night.” (P9, 30s, White, Lesbian/Gay, Cisgender)
	“It was just a waiting game. We just had to wait and stay busy and try to do other things‥” (P6, 30s, Partner, White, Hispanic, Lesbian, Transmasculine)
Asking for what one needs, knowing limits, and setting boundaries (n = 8)	“I struggled bad, but I have a co-admin on that, and she knew what was up, and I posted in the group saying, ‘Hey folks, I know people know I had a loss. Well, the due date’s here, and I’m checking in and out.’” (P2, 30s, White, Queer, Cisgender)
	“I remember one of my best friends, who’s queer, would text me every single day like ‘thinking of you, I love you,’ and at one point, I was like, ‘you have to stop. Love you, but it’s a daily reminder,’ because you don’t normally do that.” (P16, 40s, White, Queer, Cisgender)
Purposeful disclosure (n = 5)	“It’s similar to infertility. Like people don’t talk about it, and so people don’t always know that this is a very common thing. So there’s part of me that’s like, oh I should talk about it more and be open about it. But I guess I’m just not ready yet.” (P12, 30s, White, Queer, Cisgender)
	“I think we’re ready to share those stories with people who are very, very close to us that have gone through this with us, but I’m not ready to have like, my second cousin chime in on it yet.” (P11, 30s, White, Lesbian/Queer, Cisgender)
Relational Resilience (n = 16)	
LGBTQ+-specific fertility/pregnancy online groups (n = 7)	“I think knowing that I can put this out into the world with these strangers [on Facebook] and to have someone, even if it’s just a few of them to respond empathetically and share their stories back with me and give me some guidance and knowledge, that’s what I need.” (P9, 30s, White, Lesbian/Gay, Cisgender)
	“It’s [Facebook is] my whole support system at this point.” (P13, 40s, White, Bisexual/Queer, Cisgender)
Connecting specifically around pregnancy loss with other LGBTQ+ community members (n = 13)	“I saw her loss [on Facebook…. And so she commented and I reached out to her actually personally. It was so validating to know that I wasn’t alone and I wished that I’d known more about their experiences ahead of time.” (P3, 30s, White, Queer/Bi/Pan, Nonbinary)
	“It’s just something that feels… the words aren’t coming to me, but just like that shared experience. I’m being held and supported by other people that have been through it but have my similar identity and that, yeah, that have also been through the difficult journey of miscarriage.” (P10, 30s, White, Queer/Lesbian, Cisgender)
Partner support (n = 7)	“We had anticipated this possibility so my wife was able to be at the ultrasound where we found out that there was no viable pregnancy.” (P11, 30s, White, Lesbian/Queer, Cisgender)
	“[Coped with pregnancy loss] Mostly by talking to my friends and working through it with my wife.” (P6, 30s, Partner, White, Hispanic, Lesbian, Transmasculine)
In-person family/friend support (n = 3)	“I think practical support, like bringing over food. At one point a friend of mine just came over, and just sat at the table just doing her work, while I cleaned the house.” (P10, 30s, White, Queer/Lesbian, Cisgender)
Healthcare and other provider support (n = 4)	“She [the funeral home worker] was also extremely kind and she had gone through IVF and she had also lost a child before.” (P1, Black, Bisexual, Cisgender)
Collective Resilience (n = 12)	
Development and availability of LGBTQ+-specific and intersectionally-affirming support spaces (n = 3)	“I’ve since joined three or four different groups geared towards LGBT and trans people trying to conceive and with babies and breast feeding and I couldn’t find one that was good specifically about loss and miscarriage and I actually started one.” (P3, 30s, White, Queer/Bi/Pan)
	“There’s been some mention of there’s a trans-specific group of people trying to get pregnant. I can imagine for them that’s really helpful because, there again, there’s just so many other dynamics or things that are unique to that experience.” (P7, 30s, White, Queer/Lesbian/Gay, Cisgender)
Importance of LGBTQ+-specific groups for feeling seen and protected and having trust (n = 5)	“The fact that the miscarriage group and the people who supported me around miscarriages in the other groups were queer, meant that I wasn’t having to explain who I was before I explained why it was sad.” (P13, 40s, White, Bisexual/Queer, Cisgender)
	“It [LGBT Facebook Group] has been extraordinarily helpful for answering questions and learning more about the process and logistics and all of that in an affirming, welcoming space.” (P3, 30s, White, Queer/Bi/Pan, Nonbinary)
Broad commitment to share information, affirmation, and narration of underrepresented stories (n = 9)	“I also just want to modelize *[sic]* that idea of same sex couples are also allowed to get pregnant, and to want to be pregnant, and to want to have that experience. That’s something that we’re allowed to do…‥ I was wanting to normalize the idea of pregnancy as a queer experience.” (P4, 30s, White, Asexual/Biromantic, Cisgender)
	“I think that there’s a facet to the fact that yes, this is all LGBTQ folks, so there’s the same similar experience and identity but also because they’re being vulnerable and they’re sharing their stories it really, it motivates me and inspires me to share my own story.” (P10, 30s, White, Queer/Lesbian, Cisgender)

Any type of resilience endorsed by n = 17; All types of resilience endorsed by n = 11.

### Broad context of LGBTQ+ conception, pregnancy, and loss

LGBTQ+ conception, pregnancy, and loss was characterized by: 1) fear and 2) profound sadness of pregnancy loss in light of 3) unique challenges of LGBTQ+ conception, highlighting a broad context within which stigma and resilience were experienced. Some participants described significant fear that something would go wrong during their pregnancy, and then, after experiencing a loss, with subsequent pregnancies. Participants also expressed a profound sadness when their pregnancy was lost, as exemplified by P2 (30s, White, Queer, Cisgender):

*“For some weeks it was very dark*. *The most emotionally akin thing I can use to describe it’s like I sat in the bathtub and had sad playlist*. *It was like a fucking terrible breakup*, *even some of the same songs*, *because it was this process of grieving*, *and letting go*.*”*

Many participants highlighted the uniquely challenging experiences of LGBTQ+ people trying to conceive and linked that to their devastation when experiencing pregnancy loss. The medical, financial, and emotional process of LGBTQ+ conception was immense, irrespective of participant sociodemographic or socioeconomic characteristics.

*“…*. *The stakes of miscarriage for queer people can be really*, *really different… I just know so many queer people who have had a really hard time getting pregnant*, *and it’s not something that I feel like we*, *as in like people in kind of like queer communities*, *talk about openly*. *I think there is a lot of isolation often in the process…*. *I think straight people just can’t wrap their head around what it often takes for queer people to create families”*

(P10, 30s, White, Queer/Lesbian, Cisgender)

The last part of P10’s quote exemplifies participants’ overwhelming sense that heterosexual people couldn’t understand the impact of pregnancy loss on LGBTQ+ people.

*“I had one straight friend who had to do a couple of IUI’s to get pregnant with her son*, *and I just remember her being really cavalier about it*, *and just relating it to her own experience*. *I just felt like she just really did not understand because it felt like we had double the weight from having to do this in the first place*, *having to jump through all of these hoops*. *Then losing a baby just felt really awful*.*”*

(P5)

### Multi-level stigma in LGBTQ+ people’s experiences of conception, pregnancy, and loss

Stigma manifested at intrapersonal, interpersonal, and structural levels. All 17 participants endorsed at least one level of stigma, and six (35.3%) endorsed all levels of stigma at some point throughout conception, pregnancy, and/or loss.

#### Intrapersonal stigma

Subthemes highlighted through participant narratives included: 1) anticipated and internalized sexual stigma throughout pregnancy; and, 2) internalized infertility and/or pregnancy loss stigma in response to loss. Anticipated and internalized sexual stigma stemmed from negative societal perceptions about LGBTQ+ people and parenthood, specifically, from a societal belief that LGBTQ+ people should not seek biological children. As P4 (30s, White, Asexual/Biromantic, Cisgender) described:

*“I feel like even if that comes from a good place*, *there’s still a judgment that comes with that*, *of like*, *‘You’re spending your money to create more babies when you could take care of babies that are already here*.*’ I already have guilt about that …*.*”*

Some anticipated that others may perceive their loss as a consequence of or as justifiable due to their LGBTQ+ identity:

*“… I certainly had it in my mind of I don’t want people coming after me and telling me*, *“Well*, *maybe this loss happened for a reason because you shouldn’t be trying to have a child in the first place*.*”*

(P7, 30s, White, Queer/Lesbian/Gay, Cisgender)

Participants also noted internalized infertility and/or pregnancy loss stigma, some feeling shame about disclosing their pregnancy, only to subsequently experience a loss.

*“I don’t want to be identified as being the person that has infertility*.*”*

(P8, 30s, Asian and White, Asexual and Demiromantic, Cisgender)

*“I had a lot of shame around because I really early on we went [a family event]…*‥ *There was so much celebration [about the pregnancy announcement] with that and then a week later I ended up finding out that I was miscarrying…*.*”*

(P14, 30s, Latinx, Queer, Cisgender)

Ultimately, intrapersonal stigma experienced by participants was both related to pregnancy and conception as well as subsequent pregnancy losses.

#### Interpersonal stigma

Interpersonal stigma was the most pervasive and manifested through multiple mechanisms including: 1) being asked inappropriate/invasive questions; 2) receipt of unsolicited advice/judgments about conception and the pregnancy process; 3) being subjected to cisnormative and/or heteronormative assumptions; and, 4) experiencing intersecting interpersonal stigmas. As P12 (30s, White, Queer, Cisgender) described:

*“Anytime you talk about one of these issue with cis people that never have to think about this stuff*, *whether it’s the fact that I’m trying for kids or the fact that I’m trans*, *they start*, *certain subsets of people anyway ask intensely personal medical questions*, *everything from the hormones that I’m on to what surgeries I might want or not want*, *to what my genitals look like to how we have sex to who’s providing the sperm for our baby*.*”*

This participant highlighted how a common manifestation of anti-trans stigma—being asked invasive questions about one’s body—is experienced specifically during pregnancy.

Both family members and healthcare providers gave unsolicited advice about conception, explicitly judging participants for their reproductive choices and putting differential demands and expectations on LGBTQ+ people compared to heterosexual/cis people. Judgment was particularly challenging when from healthcare providers and involving multiple types of stigma. For example, P7’s (30s, White, Queer/Lesbian/Gay, Cisgender) quote exemplifies how healthcare providers’ judgment of donor choice may be influenced by HIV and sexual stigma:

*“I’ve heard of people who couldn’t even use a donor that would admit that they were a man who had sex with other men because there was this fear that that meant that that was somehow an unsafe person to use in the process*.*”*

Some participants also described intersecting marginalization from healthcare providers based on other facets of their experience, including weight, gender identity, relationship status and race. For example, P13 (40s, White, Bisexual/Queer, Cisgender), who further experienced challenges related to being a single person seeking parenthood and living rurally, highlighted weight stigma during pregnancy loss:

*“My doctor*, *literally*, *the day of the miscarriage and the day she told me that they’d do that transfer even if it was pro bono basically*, *at the same time it’s like*, *‘well*, *maybe if you take a few months off*, *the environment for the baby could be better*.*’ And I’m like*, *‘That’s code for losing weight*.*’…*. *Thank you for making it my fault that I had lost this baby*.*”*

Seemingly passive yet cisnormative and/or heteronormative assumptions fueled insensitive and harmful comments and, ultimately, made participants feel unwelcome in health and social service settings, in non-LGBTQ+-specific online spaces meant to support those using ART, and around family and friends.

*“The last time that we were going in for our IUI…the girl at the desk was like*, *‘Has your husband already made the donation*? *Is the donation here already*?*’ I was like*, *‘No*, *I’m using donor sperm*.*’”*

(P4)

Misgendering also occurred, to both masculine-of-center cisgender lesbian women as well as transmasculine and nonbinary participants. For example, P14 described misgendering among a number of interpersonal sexual stigmatizing interactions:

*“The type of things that my wife had to navigate as a masculine woman going through the trying to conceive process of accessing care*, *going to a clinic and being misgendered*. *Me being asked who I am in the room*, *am I a friend…*‥*”*

Interpersonal stigma was pervasive both offline and online. For example, a common manifestation of heternormative and cisnormative conversation on online social support groups was the emphasis on “husbands”, as P2 described:

*“I feel like straight spaces are often just pretty normative*, *like it’s a lot of the same conversations in pregnancy*. *It looks like it’s a lot of just*, *“I don’t know*, *does this one say boy to you*? *Does this one say girl to you*?*” “My husband never does the laundry*,*” and just a lot of normative shit…*.*”*

Finally, with respect to family/friends/broader community, hetornormative and/or cisnormative comments similarly reflected a general lack of knowledge about the experiences of LGBTQ+ conception:

*“My wife took our son out just to like the store when he was like a week old or something*. *And she said everyone was like*, *‘Wow*, *you look really good for just having a baby’…she was like*, *oh that was the first time anyone’s ever like- I think that was actually the only time anyone had ever assumed she had carried the baby…*‥*””*

(P12)

#### Structural stigma

Finally, structural stigma was evidenced in: 1) the embeddedness of cisnormativity and heteronormativity within systems such as pregnancy apps and the healthcare system; and, 2) the lack of LGBTQ+ specific services and resources, especially those that addressed intersecting experiences and/or are for specific sub-groups of the LGBTQ+ community. For instance, the experiences of LGBTQ+ people conceiving were institutionally erased through lack of inclusive forms:

*“We were doing this in [LGBTQ+-friendly urban center] and still a ton of their forms did not have the proper language for what we were doing…*.*”*

(P15, 30, Partner, White, Lesbian, Cisgender)

Participants were accustomed to systems designed to promote cisnormative and heteronormative assumptions, and specifically noted issues with gendering embedded within pregnancy apps.

*“It’s [pregnancy app] also very heteronormative*. *It sends me alerts like*, *‘You’re gonna be ovulating*. *You and [partner name] need to have sex*.*’…*. *It’s just default heterosexual trying to conceive*.*”*

(P4)

A lack of access to LGBTQ+ specific resources about pregnancy and about loss was also common. As P5 described:

*“I remember vividly at the time [of pregnancy loss] wanting to find resources for people who were LGBT or directed LGBT folks because I did find some books that were helpful to read*, *just to see that other people had experienced this*, *and read their experiences and feel not quite so alone*. *But all of them that I remember were visibly straight*.*”*

This erasure affected participants differentially. For example, some participants described non-gestational parents as left out of the process, which again signified a lack of understanding of LGBTQ+ families/pregnancy. There was also a lack of visibility of LGBTQ+ people of color in informational materials and/or social support/health care settings.

*“…More often than not it’s still very much a white*, *cis-het imagery*, *it’s still very much a woman and a man and a baby and they’re usually not people of color…*.*”*

(P1, Black, Bisexual, Cisgender)

Structural stigma was also evident in the near insurmountable cost and lack of access to insurance coverage for LGBTQ+ conception, noted by people from a range of socioeconomic statuses including high, as exemplified in the following quote:

*“Then to be told that IVF*, *which is so much more expensive than IUI*, *was our only option was also really difficult…*. *For someone who was having trouble making IUI work financial*, *I was just devastated…*.*”*

(P4)

Finally, a few participants noted differential standards conceiving LGBTQ+ couples had to meet, compared to heterosexual couples. In this next quote, P7 refers to stigmatizing laws around sperm donation:

*“… There are people who make the choice to say*, *‘I’m going to tell them this is my sexual partner because I don’t want to have to deal with either the medical testing that’s required*, *the psychological testing that’s required*, *the finances that go along with both of those things*, *the fact that you have to have a quarantine period of six months and then the donor has to be tested again after that period*.*’ I think a lot of it probably comes from the medical community’s still hang-up on the concept of AIDS and HIV…*.*”*

In the above statement, P7 similarly highlighted the intersection of HIV and sexual/anti-trans stigma in the way that fertility services are structured.

### Multi-level resilience in LGBTQ+ people’s experiences of conception, pregnancy, and loss

Resilience was also expressed individually, relationally, and collectively, highlighting many community strengths. All 17 participants endorsed at least one type of resilience, and 11 participants (64.7%) endorsed all types of resilience.

#### Individual resilience

In response to pregnancy loss, participants described a continuum of individual strategies from: 1) attending to their emotions in response to pregnancy grief and loss to 2) asking for what one needs, knowing limits, and setting boundaries to 3) purposeful disclosure. With respect to attending to their emotions, participants described self-soothing/distracting. Participants also used various coping strategies from remembering/honoring the loss to downplaying the significance of the loss. Moreover, many noted reflection as critical to the process of healing.

*“I did a lot of personal reflection*, *and I think in some ways*, *like really having to sit with those feelings made me process through them and deal with them in a way that I wouldn’t have had I been able to just kind of live in the space of loss and continue talking about the mechanics of it or like start those feelings about it…*.*”*

(P5)

One benefit of attending to one’s emotions was feeling ready to try to conceive again, as P14 described:

*“I just felt really proud that I finally got to a place where I could be okay with inseminating again because once the miscarriage happened I remember my first thought around it was that I don’t want to do this anymore…*. *I remember I wasn’t ready at all*, *so just getting to the point where I was finally ready to try again*, *felt like a really huge win for me*.*”*

Asking for what one needed and setting boundaries when anticipated supports (e.g., online spaces) were not helpful was an important part of recovery from loss, an approach that typically helped participants. This was demonstrated by P8: *“I visited the miscarriage sub-reddit*, *and decided it was really depressing*, *and then I left*.*”*

For some participants, not sharing about their loss was protective and intentional, both from the perspective of protecting against anticipated anti-trans and/or sexual stigma, as exemplified in the quote by P12: *“If I don’t put it out there*, *then nobody can say the wrong thing back*.*”* However, purposeful disclosure also protected against potential pain related to vulnerability of sharing with unknown others online, as was the case with P14: *“It just felt too intimate and too much information for me to wanna share with just everybody”* or, as was the case with P7, honored a participant’s mental space:

*“I’m still in this protective space of where I am in the process right now*. *And then eventually when I get to the point where I can be very open about it*, *I’ll share those things*.*”*

#### Relational resilience

Relational resilience was noted through the process of: 1) seeking out and engaging with LGBTQ+-specific online fertility and pregnancy groups; 2) connecting with others who have experienced pregnancy loss; 3) partner interactions; and, 4) healthcare provider interactions. A cluster of LGBTQ+-specific Facebook groups were key to fostering relational resilience among participants.

*“I need support*. *I need my community*. *These people that I’m connected with are that*… *It was just a wonderful support system*, *and then they were there cheering me on*, *the same people when I was ready to try again…*.*”*

(P2)

Recognition and relationship building happened between Facebook group members, as participants became invested in each other’s journeys. While participants recognized the emotional labor of relating to others about pregnancy loss, it was worth it in terms of the acceptance, understanding—and tangible information—gained, particularly in the absence of LGBTQ+-specific materials:

*“Then we started having things go wrong*, *I was looking [Facebook group] to see is this normal*? *How worried should I be*?*…”*

(P8)

Hearing others’ stories online also facilitated hope, reduced pain, resulted in social support, and ultimately, reduced internalized pregnancy loss stigma, as P16 (40s, White, Queer, Cisgender) described:

*“I think it’s helpful in the sense that it’s kind of confronting that stigma*, *that*, *you know*, *this happened to someone you know relatively well and you wouldn’t judge them so why are you judging yourself kind of thing…*.*”*

Multiple participants described the support received within their intimate partnership, though some recognized the limitations to the understanding from their non-gestational partner.

*“I gave my partner a big hug and it was a good experience in that moment despite what was happening*.*”*

(P1)

Finally, some participants expressed relational resilience through meaningful relationships with health and other care providers.

*“I had a wonderful doctor who was understanding and took care of me and respected my gender and all that*. *I needed that in that moment*. *I needed to not be misgendered on the worst day of my life and I had that*.*”*

(P3, 30s, White, Queer/Bi/Pan, Nonbinary)

#### Collective resilience

Collective resilience was seen in participant narratives through their rich descriptions of 1) the development and availability of LGBTQ+-specific and intersectionally-affirming support spaces; 2) the importance of LGBTQ+-specific groups for feeling seen and protected and having trust; and, 3) their broad commitment to share information, affirmation, and narration of underrepresented stories of loss. Several participants talked about developing identity-specific LGBTQ+ fertility/pregnancy resources. For example, as P1 described:

*“So*, *I’m just really intentional about that so I really wanted that to be part of it and I also just really wanted it to be like the blog*, *I just really wanted it to be really black to be honest*.*”*

Overall, online LGBTQ+-specific groups were incredibly important for feeling seen as an LGBTQ+ person, protected from anti-trans and sexual stigma as well as cisnormativity and heteronormativity, and to have trust in information shared.

*“I don’t think there’s one straight or heterosexual group that I’m a part of*. *They’re all queer people…*. *There’s enough heteronormative*, *heterosexual*… *Whatever it is*, *heteronormativeness in my life that*, *this one little aspect that I can control*, *I don’t have to*.*”*

(P17, 30s, Partner, Unidentified race, Queer, Cisgender)

Finally, most participants noted the importance of sharing their experiences for the purpose of broadening representation of LGBTQ+ conception/pregnancy/parenting experiences, in both online and offline spaces.

*“That group at the [community center] is something I hope one day when I become a parent I can go back and talk to that group and be the person who shared about their experiences because I think that there’s less information out there*, *I think*, *for LGBT people…*. *For the queer people that I’m friends with on Facebook*, *I want them to see this is possible if you want to do this…*. *Also*, *for the straight people who will see that*, *also to be able to see this is possible*.*”*

(P7)

## Discussion

By applying minority stress theory [24–27] to understand LGBTQ+ peoples’ experiences of conception, pregnancy, and pregnancy loss, we identified minority stressors of intrapersonal, interpersonal, and structural stigmas as well as individual, relational, and collective resilience in both offline and online spaces, which may mitigate the negative impacts of these stigmas. These findings can be leveraged to identify recommendations for strengths-based and affirming approaches to reproductive healthcare for LGBTQ+ people.

Participants described some experiences of pregnancy loss stigma prevalent among cisgender and heterosexual women (e.g., self-blame/shame) [12, 13]. However, feelings of self-blame/shame for our participants were exacerbated within a context whereby they feared others would think their loss justified or even warranted due to their LGBTQ+ identity. In this way, our findings underscored how internalized pregnancy loss stigma intersected with anti-trans and sexual stigma at intra- and interpersonal levels, specifically within the pregnancy process. For example, findings underscored the complexity of navigating pregnancy as an LGBTQ+ person, where one is simultaneously subjected to patriarchal standards and expectations of (biological) reproduction, and heteronormative and cisnormative positions of how one should become a parent (by adopting).

In light of interpersonal stigma from healthcare providers, we join prior scholars who have called for more culturally-sensitive reproductive healthcare and the explicit addressing of sexual and anti-trans stigma [3, 33, 42, 64]. Provider-level training to reduce biases can reduce negative attitudes towards LGBTQ+ people [65]. Training and education could include: 1) how cis/heteronormativity influence provider manifestations of sexual and anti-trans stigma; 2) how these stigmas can be damaging to the health and wellbeing of LGBTQ+ people during pregnancy; 3) the use of affirming language, and the un-gendering of language associated with pregnancy; and, 4) experiences of pregnancy loss for LGBTQ+ people. Our findings further corroborate prior work recommending training not only for healthcare providers, but all people who interact with patients, including front desk staff [66]. We extend past work by recognizing other settings in which LGBTQ+ affirming training is needed, such as sperm banks.

As recently as a decade ago there were no standard texts that included information about care LGBTQ+ people, and numerous studies and reviews of health issues have documented a continued gap in health care education [67–69]. In one study, 80% of nurses surveyed reported that they had no education on lesbian, gay, bisexual, transgender, queer or questioning (LGBTQ+) issues. As a result, it is necessary to embed additional training in health care training programs that normalizes experiences LGBTQ+ conception, pregnancy, and pregnancy loss and fosters providers’ clinical skills to provide support to LGBTQ+ people during pregnancy [42, 70, 71].

Erasure of LGBTQ+ peoples’ experiences of conception, pregnancy, and pregnancy loss were described, whereby there was a lack of materials, services, and policies and practices embedded organizationally to address the needs of this group. At an organizational level, anti-discrimination policy that takes into consideration intersecting experiences may be useful to set a tone for the provision of affirming care [72]. Forms—either paper or electronic—should be updated to be inclusive of conception choices and pregnancy experiences of LGBTQ+ people. Materials specific to pregnancy loss are urgently needed, especially those specific to LGBTQ+ people [33]. These materials should be intersectionally-affirming, inclusive of LGBTQ+ people of different abilities, ethnicities, sexual orientations, and gender identities [23]. Organizations should develop and/or provide options for support, grief, and coping with loss, both online and offline. At an even broader level, policy changes are needed that address differential access to conception among LGBTQ+ compared to cisgender/heterosexual persons, and increase the affordability of ART, which is a major stressor not just for LGBTQ+ people but for many [23, 73, 74].

Ultimately, federal human rights protections are necessary to reduce physical and mental health disparities among LGBTQ+ people [75, 76]. Future research may extend research on sexual orientation inequities in pregnancy and birth outcomes [3, 5] to also understand gender inequities in such outcomes and pathways between stigma and negative birth outcomes for LGBTQ+ people at structural (e.g., lack of access to healthcare), interpersonal (lack of access to social support) and intrapersonal/biological levels (allostatic load) [29, 30]. These pathways should be explored in reference to the resultant mental health impacts of pregnancy loss. Throughout this discussion we have emphasized the role of providers, organizations, and policy makers to reduce stigmatization of LGBTQ+ people, rather than place the emphasis on how LGBTQ+ people can build resilience. In this way, we seek to promote structural change rather than personal adaptation to stigma. However, in the interim, given the pervasiveness of stigma, and that resilience can reduce the negative impacts of sexual stigma, including biological/physiological impacts of allostatic load [50], strategies to increase resilience should be further explored in future work, in order to reduce the traumatization of LGBTQ+ people during pregnancy and in particular during pregnancy loss.

While our findings showed that LGBTQ+ people are strong individually in the context of stigma and pregnancy loss, relational and collective resilience of participants resonated strongly. We build on previous work describing LGBTQ+ peoples’ resilience in the context of pregnancy loss [23] to further elucidate a multi-level (individual, relational, and collective) theoretical conceptualization of resilience. Our findings corroborate previous studies conducted with LGBTQ+ people that online spaces can be a significant source of social support [51, 52], and extend findings about online social support for cisgender heterosexual women who have experienced pregnancy loss [53] to account for LGBTQ+ peoples’ specific experiences. Future studies may draw on human-computer interaction literature to further theorize how social technologies can promote resilience [77, 78]. Healthcare providers may recommend online LGBTQ+ support groups as a legitimate source of social support for LGBTQ+ people trying to conceive, particularly given prior evidence that in-person social support groups are not always inclusive or affirming of LGBTQ+ people who have experienced pregnancy loss [23].

Our study is limited by the homogeneity of our sample, which included predominantly white, cisgender, partnered, urban women of high socioeconomic status. It is quite likely that the sample composition was influenced by the use of our social media networks as a method of recruitment, whereby we reached a select sample of those holding more privileged identities and those more likely to participate in research (e.g., those with PhDs). However, given that we also recruited from the largest LGBTQ+ parenting groups on Facebook, future attention is needed to understand if these groups are also exclusive of those holding more marginalized socioeconomic statuses, and to develop best practices to recruit participants who have access to less resources and thereby more barriers to conception and pregnancy loss support. Studies have examined sample characteristic differences among those recruited into sexual and reproductive health research via in-person versus social media methods [79]. Future studies should use broader recruitment methods and additional community recruitment strategies to generate a more generalizable sample. Moreover, future qualitative studies could use additional strategies to promote rigor in qualitative research, such as assessing inter-rater reliability and engaging in member checking. Despite this limitation, we provided a preliminary exploration of the experiences of non-gestational parents, people of color, trans/nonbinary persons, and those who are single, addressing gaps in a literature predominantly focused on cisgender sexual minority women [3, 5, 14, 15, 64, 80–82]. However, more diversity of participants is needed in future research and these study results must be interpreted with caution, particularly with respect to interpretations of the experiences of trans and non-gestational partners. While our non-gestational partner participants (n = 3) shared narratives reflective of and similar to those having physically experienced a loss (n = 14), a larger sample size of non-gestational partners may have identified more nuanced experiences.

An intersectional approach may facilitate understanding inequitable access to reproductive healthcare that occur within the LGBTQ+ community, due to intersecting oppressions [23, 45]. While there has been some recent recognition of the fertility and reproduction experiences of trans people [83, 84] widespread research is severely limited and few studies have focused on pregnancy loss [6, 42]. Future studies conducted from a stigma framework should continue to document the experiences of specific sub-groups of the LGBTQ+ community. Finally, given the nuances of both structure of the healthcare system and laws/policies related to LGBTQ+ human rights, results can only be generalized to those in a U.S. context. It is possible that LGBTQ+ people living in settings with more consistent LGBTQ+ protections and/or universal healthcare coverage may report different experiences of stigma and resilience in the process of conception, pregnancy, and loss.

## Conclusion

This qualitative study highlighted the complexity of conception, pregnancy, and pregnancy loss among LGTBQ+ people in light of multi-level stigmas experienced on their journeys to parenthood. Despite negative interpersonal interactions and structural barriers to accessing parenthood, and devastating experiences of pregnancy loss, our participants showed their individual, relational, and collective strength. Structural changes at the provider-, organizational-, and policy-level are critical to foster health and wellbeing of LGBTQ+ people during pregnancy and pregnancy loss. This work contributes to scholarship and practice for more inclusive interactions for LGBTQ+ people in diverse pregnancy journeys online and off. While that important work is ongoing, LGBTQ+ people may foster resilience through engaging with other LGBTQ+ people in offline and online spaces.
